# Controlling interlayer excitons in MoS_2_ layers grown by chemical vapor deposition

**DOI:** 10.1038/s41467-020-16023-z

**Published:** 2020-05-13

**Authors:** Ioannis Paradisanos, Shivangi Shree, Antony George, Nadine Leisgang, Cedric Robert, Kenji Watanabe, Takashi Taniguchi, Richard J. Warburton, Andrey Turchanin, Xavier Marie, Iann C. Gerber, Bernhard Urbaszek

**Affiliations:** 1 Université de Toulouse, INSA-CNRS-UPS, LPCNO, 135 Avenue Rangueil, 31077 Toulouse, France; 2 Institute of Physical Chemistry, Friedrich Schiller University Jena, 07743 Jena, Germany; 3 Department of Physics, University of Basel, Basel, Switzerland; 4 National Institute for Materials Science, Tsukuba, 305-0044 Ibaraki Japan; 5 Abbe Centre of Photonics, 07745 Jena, Germany

**Keywords:** Two-dimensional materials, Two-dimensional materials

## Abstract

Combining MoS_2_ monolayers to form multilayers allows to access new functionalities. Deterministic assembly of large area van der Waals structures requires concrete indicators of successful interlayer coupling in bilayers grown by chemical vapor deposition. In this work, we examine the correlation between the stacking order and the interlayer coupling of valence states in both as-grown MoS_2_ homobilayer samples and in artificially stacked bilayers from monolayers, all grown by chemical vapor deposition. We show that hole delocalization over the bilayer is only allowed in 2H stacking and results in strong interlayer exciton absorption and also in a larger A-B exciton separation as compared to 3R bilayers. Comparing 2H and 3R reflectivity spectra allows to extract an interlayer coupling energy of about *t*_⊥_ = 49 meV. Beyond DFT calculations including excitonic effects confirm signatures of efficient interlayer coupling for 2H stacking in agreement with our experiments.

## Abstract (web summary)

The authors investigate the interplay between the stacking order and the interlayer coupling in MoS_2_ homobilayers as well as artificially stacked bilayers grown by chemical vapour deposition, and identify the interlayer exciton absorption and A-B exciton separation as indicators for interlayer coupling.

## Introduction

Transition metal dichalcogenides (TMDs) with the form MX_2_ (M = Mo, W, Ti, etc. and X = S, Se, Te) have tunable electronic properties from metallic to semiconducting depending on the crystal symmetry, composition, and number of layers^1–9^. The band structure of TMD semiconductors is drastically modified by changing the sample thickness by just one atomic monolayer^10–12^. For instance, the combination of two different monolayer materials such as MoSe_2_–WSe_2_ into a heterobilayer results in type II band alignment and opens new research perspectives on periodic moiré potentials for carriers in the different layers and the resulting interlayer excitons^13–17^. Twisted homobilayers of graphene, WSe_2_, and MoSe_2_ allow accessing new superconducting phases and correlated insulating states^18–20^. To access the new functionalities provided by assembling monolayers to form multilayers it is necessary to identify physical parameters that strongly depend on interlayer coupling and to experimentally control them. One approach is to compare chemical vapor deposition (CVD)-grown MoS_2_ bilayers with artificially stacked bilayers made from CVD monolayers with 2H (180° twist angle) and 3R (0° twist angle) stacking. Studying these two precise alignments is also relevant for samples initially assembled with other twist angles as reconstruction results also in these experiments in the formation of μm-wide 2H and 3R areas^21,22^, which are energetically most stable. To artificially stack large area CVD layers and control interlayer coupling through stacking (i.e., 0° or 180° twist angle) is technologically relevant for 2D materials optoelectronics^17^, as CVD substrates are covered by a large number of monolayers and are very practical to stack (twist) due to their symmetric triangular shape and well-characterized edge termination.

Here we show that the valence states for 2H bilayers are strongly impacted by interlayer coupling as the hole is delocalized over the two layers^23–25^. This results in important changes in the optical spectra governed by K–K transitions as we observe strong absorption from interlayer excitons and a clear change in separation between A- to B-exciton transition in differential white light reflection at *T* = 4 K. These observations are made possible due to the drastically improved optical quality of CVD samples removed from the growth substrate and encapsulated in hBN^26^. We show that both indicators for interlayer coupling are absent in the measured 3R bilayer spectra as a hole hopping between the layers is symmetry forbidden^27^. Comparing for 3R (no interlayer coupling) and for 2H the A–B exciton absorption spectra allows us to extract an experimental value of the perpendicular hopping (coupling) term of *t*_⊥_ ≈ 49 meV, important for moiré superlattices^28^ and so far only roughly estimated from theory^27^.

In addition to our optical spectroscopy experiments we show in density functional theory (DFT) calculations, as well as by applying GW-type approaches, that the valence band (VB) splittings for 2H as compared to 3R are different due to interlayer coupling. In our calculated absorption spectra, including excitonic effects by solving the Bethe–Salpeter-equation (BSE) on top of GW calculations, for 2H stacking we show strong interlayer exciton absorption, absent for 3R stacking.

## Results

### Interlayer excitons in as-grown CVD MoS_2_ homobilayers

The thermodynamically most stable configurations of TMD homobilayers are the 2H and the 3R stacking^24,30^. In practice, most naturally occurring molybdenite shows 2H, not 3R stacking. In this work, we focus on high-quality CVD-grown flakes for several reasons: during CVD growth of MoS_2_ both 2H and 3R stackings for bilayers can occur^31^ and we are therefore able to compare the optical response for samples grown under identical conditions. Secondly, as all CVD flakes on our substrate show the same edge termination, we can artificially stack two layers in 2H and 3R configuration with a precise twist angle to compare with the as-grown samples—see “Discussion” below. Third, many monolayers cover the SiO_2_ substrate and can all be picked up in a single step, which makes fabrication of bilayer structures very efficient. CVD flakes with a larger surface area are also more convenient to fabricate devices with electrical contacts.

**Fig. 1:**
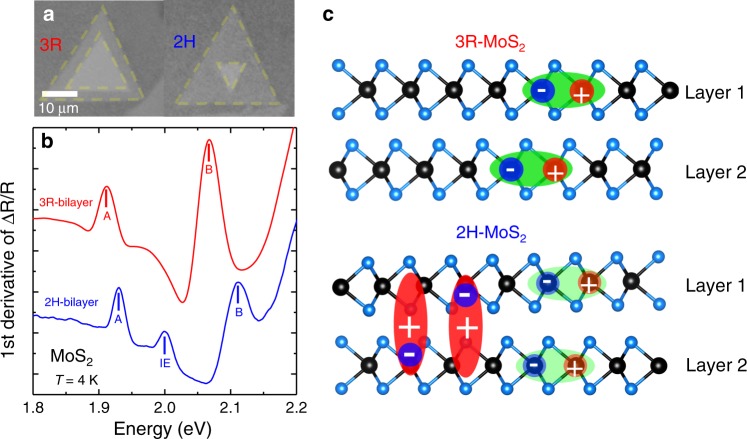
Spectroscopy of as-grown CVD bilayers encapsulated in hBN. **a** Optical microscope images of as-grown 3R (left) and 2H CVD MoS_2_ bilayers (right) on SiO_2_/Si before pick-up. **b** First derivative of white light reflection spectrum for as-grown 2H-bilayer (blue) and as-grown 3R-bilayer (red), recorded at *T* = 4 K, both bilayers are encapsulated in high-quality hBN for optical spectroscopy^29^. **c** Schematic of 3R stacked bilayer with intralayer excitons (top) compared to 2H stacked bilayer where in addition interlayer excitons are observed as in panel (**b**).

Optical microscope images of as-grown CVD bilayers on SiO_2_/Si with 3R and 2H stacking are presented in Fig. 1a. The stacking can be determined already by the relative rotation of the triangular monolayers and is confirmed in second harmonic generation (SHG) experiments^33,34^. The SHG signal for 2H stacking was not detectable (inversion symmetry restored) but we could perform detailed angle-dependent SHG for the 3R stacking (broken inversion symmetry), see Supplementary Figs. 1 and 2. The high-quality MoS_2_ bilayers and monolayers were grown by a modified CVD process in which a Knudsen-type effusion cell is used for the delivery of sulfur precursor^35^. Using a water-assisted pick-up technique^32^, the as-grown CVD bilayers have been deterministically transferred and encapsulated in hBN to achieve high optical quality^26^, which has recently been shown to be crucial for optical spectroscopy on CVD samples lowering the typical emission linewidth from about 50 meV to below 5 meV at T = 4 K. An example of a photoluminescence spectrum of a CVD MoS_2_ monolayer used here is shown in Supplementary Fig. 3. The thickness of the top and bottom hBN has been carefully selected to optimize the oscillator strength of the interlayer excitons (IEs)^24^. After encapsulation, the samples were cooled down to *T* = 4 K in a closed-cycle cryostat and a series of differential reflectivity measurements with a home-built confocal microscope have been performed at different locations of the samples, see “Methods”. We define differential reflectivity as (*R*_sam_ − *R*_sub_)/*R*_sub_, where *R*_sam_ is the intensity reflection coefficient of the sample with the MoS_2_ layers and *R*_sub_ is the same structure without the MoS_2_. Note that the overall shape and amplitude of the differential reflectivity signal also depends on cavity effects (thin-layer interference) given by top and bottom hBN and SiO_2_ layer thickness (see ref. ^36^ for details). In Fig. 1b, the first derivative of the differential reflectivity spectra for as-grown CVD 2H and 3R MoS_2_ bilayers can be compared, see Supplementary Fig. 5 for differential reflection spectra. There are two striking differences between the 2H and 3R bilayer spectra: (i) While A and B intralayer excitons are identified for both configurations, a pronounced feature at ≈2 eV appears exclusively in the 2H bilayer. This feature is assigned to an interlayer state, its energy being in good agreement with the very recently identified IEs in high quality and hBN encapsulated, exfoliated MoS_2_ bilayers with 2H stacking^23,24,37^, in contrast to CVD-grown samples studied here. This observation of IEs is made possible by our specific CVD sample preparation for the optical spectroscopy experiment^26^. IE absorption was not detectable in very detailed earlier works due to considerably larger optical linewidth or detection of emission and not absorption^38–41^. In contrast to 2H stacking, in the 3R configuration no additional states are detected between the A- and B-excitons, thus indicating that the delocalization of holes is not allowed in this particular stacking order^24^, see below for a more detailed discussion. (ii) The separation between the A- and B-exciton transitions is considerably larger in the 2H bilayers (about 185 meV) as compared to the 3R bilayer (about 150 meV, mainly given by the spin–orbit splitting in the VBs). This is a second indication for efficient interlayer coupling of A–B valence states for 2H stacking, as the separation of the valence states mainly governs the A–B exciton separation^23,27,42^.

### Controlling interlayer coupling through stacking

**Fig. 2:**
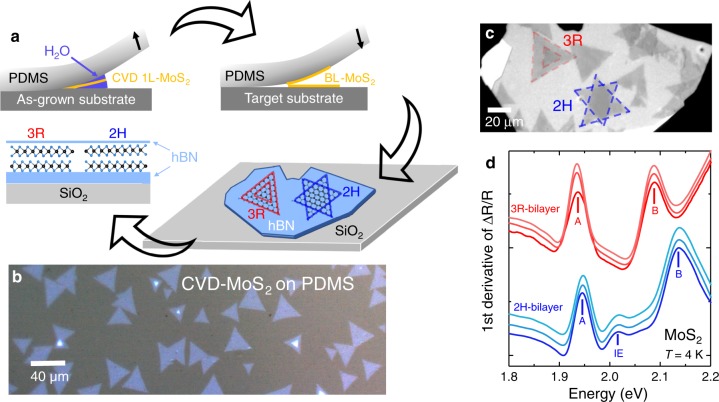
Artificial stacking of CVD monolayers into bilayers. **a** Schematic of sample pick-up, bilayer assembly, and encapsulation for optics. **b** Optical micrograph of CVD-grown MoS_2_ monolayers and a few homobilayers, transferred to the PDMS stamp following water-assisted pick-up^32^ from the growth substrate. **c** Artificially-assembled 3R and 2H MoS_2_ homobilayers, fabricated by an all-dry deterministic transfer process. **d** First derivative of reflectivity spectra collected from three different areas of the artificially stacked 3R (red) and 2H (blue) MoS_2_ homobilayers, shown in (**c**). Spectra have been shifted for clarity.

In Fig. 1 we show that as-grown CVD MoS_2_ bilayers experience interlayer coupling resulting in interlayer exciton formation, here observed for a non-contaminated interface between the top and bottom layer. Contamination from secondary transfer processes could potentially suppress the coupling between the layers and hence IE formation. By choosing 2H or 3R orientation manually when stacking CVD-grown monolayers to form bilayers one can allow or disallow hole tunneling between the layers, respectively. This requires to pick-up the CVD-grown monolayers from their growth substrate while maintaining their structural integrity, optical quality, and a sufficiently clean interface after transfer. Furthermore, fine control of the twist angle between the top and bottom layer is needed, since the IE formation is allowed only in a precise stacking order, see sample preparation schematic in Fig. 2a. Here we use water-assisted deterministic transfer that allows the ability to controllably assemble CVD bilayers with the desired twist angle^32^. First, CVD-grown monolayers have been carefully picked up from the growth substrate and transferred to polydimethylsiloxane (PDMS) (Fig. 2b)^32,43^. The structural integrity of the CVD-grown monolayers is preserved in this case and the following step is to slowly assemble 2H and 3R bilayers and encapsulate them in hBN as shown in Fig. 2c. Small deviations from 0° or 180° twist angle are expected but natural reconstruction of the bilayer will again favor the lowest energy arrangement, 3R and 2H, respectively^21,22^.

Differential reflectivity spectra have been collected from 10 different areas of the assembled 2H and 3R bilayers of Fig. 2c. In Fig. 2d, three typical examples of the assembled 2H and 3R spectra are presented. The spectra show a striking resemblance with the as-grown bilayer spectra discussed before in Fig. 1b. So also for the assembled 2H bilayers we identify clear interlayer exciton absorption and an increased separation between the A- and B-excitons. It is important to note that the IE transition was clearly observed over the whole surface area of the manually constructed 2H bilayer. We take it as a strong indication of efficient interlayer coupling and possibly efficient reconstruction/self-rotation to the 2H configuration. We therefore further confirm the formation of IEs exclusively in the 2H stacking. By manually choosing the stacking configuration i.e., twist angle, it is possible to tune the VB splitting and the formation of interlayer excitons in a large area, high-quality CVD samples.

### Beyond-DFT band structure calculations including GW+BSE

**Fig. 3:**
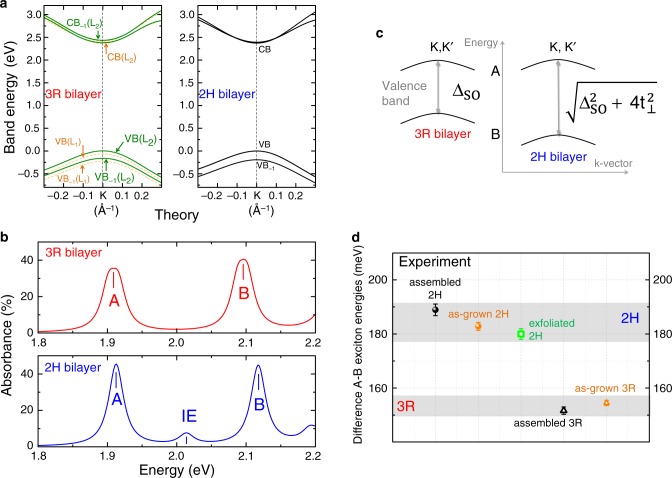
Interlayer coupling in theory and experiment. **a** Valence and conduction bands around K-point calculated at the *G*_0_*W*_0_ level for 2H and 3R stacking, with the energy value of the VB set to 0 in K. **b** Calculated absorption using *G*_0_*W*_0_+BSE approach for both stackings, see “Methods” for the computational details. Complete band structures are given in Supplementary Fig. 4. **c** Schematic of the A- and B-valence bands for 3R bilayers (left) and 2H bilayers (right) as a function of the spin–orbit splitting Δ_SO_ and the interlayer coupling parameter *t*_⊥_. **d** Energy difference between B- and A-exciton for the as-grown (orange), as well as artificially-assembled (black) 2H and 3R MoS_2_ homobilayers. The error bars represent the standard deviation extracted over 10 different spectra in each case. Gray shaded area is a guide to the eye to underline clear differences between 3R and 2H bilayers.

In addition to optical spectroscopy we perform beyond DFT calculations to study the striking differences between 2H and 3R MoS_2_ bilayers, see “Methods” for the computational details. Please note that our GW+BSE calculations are performed for MoS_2_ bilayers in vacuum for simplicity and not in hBN. The general target of our calculations is to understand the microscopic origin of the optical transitions and to reproduce the energetic order qualitatively. In GW calculations we compare band structures corrected by screening effects, the exciton description being added later. In the vicinity of the K-point of the Brillouin zone, see Fig. 3a and schematic in Fig. 3c, differences between 2H and 3R stacking in VB and VB_−1_ states are clear. We find a VB splitting in the 2H bilayer that is 19 meV larger than in the 3R bilayer. By solving the BSE we obtain the absorption for the 2H and 3R bilayers shown in Fig. 3b. The main characteristics are (i) the presence of a strong interlayer exciton peak in 2H and (ii) a larger A–B exciton separation for 2H than for 3R configuration, exactly as found in the experiments in Figs. 1 and 2. Interestingly, in 3R stacking, there is a minor departure from degeneracy that splits VB and VB_−1_ states from distinct layers when they remain degenerate in 2H configuration. As a consequence, two distinct A-type excitons separated by only 14 meV constitute the A-peak of the 3R-bilayer absorption spectrum in our calculation, explaining its larger width compared to the 2H case, see Fig. 3c. The origin of this splitting, that keeps VB(L1) and VB(L2) states to be of the same spin-states, is the lack of symmetry inversion combined with different atomic environments for Mo atoms^44^. This is especially true for the VBs since the atomic orbitals that constitute those states are particularly sensitive to crystal field splitting.

## Discussion

To summarize the main experimental findings, first we observe strong interlayer exciton absorption between the main A- and B-exciton transitions for CVD-grown (Fig. 1b) and artificially stacked (Fig. 2d) 2H bilayers, whereas this interlayer transition is absent for 3R stacking as hole tunneling is symmetry forbidden and only intralayer exciton transitions are observed. A second striking observation is that the stacking of the layers also affects the energy difference between A- and B-exciton transitions. This is demonstrated in Fig. 3d, where the A–B exciton energy difference is compared between as-grown and assembled 2H and 3R bilayers. It is apparent that 2H bilayers exhibit a significantly larger energy difference between the A- and B-exciton states, compared to 3R bilayers.

Our next target is to experimentally extract the interlayer hopping term, *t*_⊥_ based on a k·p model of bilayers in the vicinity of K points^23,27,28^ and compare it to post-DFT estimates. As indicated in Fig. 3c, for 3R stacking the measured A- to B-exciton splitting S_3R_ is roughly given by the spin–orbit splitting as S_3R_ = Δ_SO_. For 2H-stacking the A–B splitting in the valence band depends on the coupling energy *t*_⊥_ as ${}\mathrm{S}_{\text{2H}}=\sqrt{{\Delta }_{\text{SO}\,}^{2}+4{t}_{\perp }^{2}}$ and hence

1 $${t}_{\perp }=\sqrt{\frac{{\,\text{S}}_{\text{2H}}^{2}-{\Delta }_{\text{SO}\,}^{2}}{4}},$$

where S_2H_ is the measured A–B exciton splitting of the as-grown 2H MoS_2_ bilayer and as a value for Δ_SO_ we take the measured A–B separation in the 3R sample. For S_2H_ = 183 meV and Δ_SO_ = 155 meV, we obtain *t*_⊥_ ≈ 49 meV. This value can be compared to the ones extracted from our standard DFT calculations as previously done^27^, or from more advanced GW and GW+BSE calculations. Table 1 summarizes calculated valence band splittings and A–B energy differences for monolayer, 2H and 3R stacking as well as the corresponding coupling strength, directly extracted from the VB splitting as in ref. ^28^ or Eq. (1). The agreement between theoretical, including previous rough estimates^27^, and experimental results is good as we reproduce the larger A–B splitting for the 2H bilayers as compared to 3R. Although we measure exciton transitions and not directly the VB splitting in the 2H bilayer, the agreement between theory and experiment strongly supports our interpretation for the reason behind the different A–B exciton splitting. It should be noted that for 3*R* bilayers, *t*_⊥_ = 0 since interlayer hopping is not allowed in this case. This short numerical analysis highlights that the efficiency of this interlayer coupling will depend on the ratio of *t*_⊥_ versus the spin–orbit VB splitting^45^, which is much smaller in MoS_2_ (Δ_SO_ ≈ 150 meV) as compared to WSe_2_ (Δ_SO_ ≈ 430 meV). This leads in principle to tunable interlayer coupling for MoS_2_^46^ and so-called spin-layer-locking for WSe_2_ bilayers^47^.

**Table 1:** Valence band splittings, A–B transition energy differences (S) extracted from GW and GW+BSE calculations and the corresponding interlayer coupling parameters.

	Monolayer	3R-bilayer	2H-bilayer	*t*_⊥_
VB splitting	178 (189)	175 (189)	194 (203)	57 (42)
S	185	186	205	43

Values extracted for standard DFT calculations are in parentheses. All values are given in meV.

Larger A–B exciton splitting in bulk MoS_2_ for 2H compared to 3R stacking has also been demonstrated in early experiments using optical transmission spectroscopy^48,49^ and more recently in angle-resolved electron emission spectroscopy^50^. The A–B exciton splitting in MoS_2_ bilayers has been previously studied by several groups^38,51–54^. In these reports, the spin–orbit coupling and interlayer coupling have been discussed, but neither interlayer exciton formation nor an experimental analysis of the coupling term *t*_⊥_. In our study, the direct comparison in the same set-up on the same substrate of 2H and 3R bilayers with good optical quality due to encapsulation allows to determine the difference in A–B exciton energies precisely, which we ascribe to interlayer coupling, as supported by our quasi-particle GW and absorption spectrum including excitonic effects calculations. From an experimental point of view, our results suggest two practical test criteria for interlayer coupling following artificial stacking: the strong interlayer exciton absorption and the clear difference in A–B exciton transition energies. The physics discussed here for 2H and 3R bilayers is also relevant for samples with a twist angle slightly different from 0° or 180° as reconstruction/self-rotation results in artificial stacks typically in large areas of 2H and 3R stacking, which will show the optical properties of the samples investigated here. Interestingly, interlayer exciton absorption has been reported in bulk 2H-MoTe_2_ and bulk 2H-MoSe_2_^55,56^. Please see ref. ^57^ for related work on bilayer MoS_2 _.

## Methods

### CVD samples growth

MoS_2_ crystals were grown on thermally oxidized silicon substrates (Siltronix, oxide thickness 300 nm, roughness  <0.2 nm RMS) by a modified CVD growth method in which a Knudsen-type effusion cell is used for the delivery of sulfur precursor^35^.

### Sample pick-up and encapsulation

The SiO_2_/Si substrate for hosting the van der Waals structure was cleaned with a 10-min ultrasonication bath in acetone and isopropanol followed by oxygen plasma exposure. A clean PDMS stamp was first placed on a glass slide and the SiO_2_/Si substrate containing the as-grown CVD MoS_2_ monolayers and homobilayers was brought in contact with the PDMS stamp^32^. The substrate was pressed against the PDMS stamp and distilled water droplets were injected at the perimeter of the substrate. Water droplets penetrated into the SiO_2_/MoS_2_/PDMS interface and after 1 min the SiO_2_/Si substrate was carefully lifted, resulting into the transfer of a large area of CVD-grown MoS_2_ triangles onto the PDMS stamp, as shown in Fig. 2b. During the water-assisted pick-up technique, high-purity (>20 MΩ) deionized water was used and the samples were dried with a nitrogen gun. Finally, hBN flakes were exfoliated from high-quality bulk crystal^29^ onto the target substrate and subsequent deterministic-dry transfer of the CVD-grown MoS_2_ triangles from the PDMS stamp on top of the hBN was applied. Thermal annealing at 150° for 30 min is performed after each transfer step.

### Optical spectroscopy set-up

Low-temperature reflectance measurements were performed in a home-built micro-spectroscopy set-up assembled around a closed-cycle, low vibration attoDry cryostat with a temperature controller (*T* = 4–300 K). The white light source for reflectivity is a halogen lamp with a stabilized power supply focussed initially on a pin-hole that is imaged on the sample. The emitted and/or reflected light  is dispersed in a spectrometer and detected by a Si-CCD camera. The excitation/detection spot diameter is  ≈1 μm, i.e., smaller than the typical size of the homobilayers.

### Methods for DFT and GW calculations

The atomic structures, the quasi-particle band structures, and optical spectra have been obtained from DFT calculations using the VASP package^58,59^. The projector-augmented wave scheme^60,61^ has been used to treat core electrons. Motivated by the fact that 2H and 3R bilayers have very similar lattice parameters^62^ and since upon geometry optimization on MoS_2_ monolayers the obtained lattice parameter is 3.22 Å, we have kept this value for all the runs. No significant change in the interlayer distance, as defined here by the separation between the two parallel planes containing Mo atoms, is observed when passing from 2H to 3R configuration, i.e., 6.17 and 6.13 Å respectively. A grid of 15 × 15 × 1 k-points has been used, in conjunction with a vacuum height of 21.9 Å, for all the calculation cells. The geometry’s optimization process has been performed at the PBE-D3 level^30^ in order to include van der Waals interaction between layers. All the atoms were allowed to relax with a force convergence criterion below 0.005 eV/Å. Heyd–Scuseria–Ernzerhof (HSE) hybrid functional^63–65^ has been used as an approximation of the exchange-correlation electronic term, including SOC, to determine eigenvalues and wave functions as input for the full-frequency-dependent GW calculations^66^ performed at the *G*_0_*W*_0_ level. An energy cutoff of 400 eV and a Gaussian smearing of 0.05 eV width have been chosen for partial occupancies, when a tight electronic minimization tolerance of 10^−8^ eV was set to determine with a good precision the corresponding derivative of the orbitals with respect to *k* needed in quasi-particle band structure calculations. The total number of states included in the GW procedure is set to 1280, in conjunction with an energy cutoff of 100 eV for the response function, after a careful check of the direct bandgap convergence (smaller than 0.1 eV as a function of k-points sampling). Band structures have been obtained after a Wannier interpolation procedure performed by the WANNIER90 program^67^. All optical excitonic transitions have been calculated by solving the Bethe–Salpeter equation^68,69^, using the 12 highest valence bands and the 16 lowest conduction bands to obtain eigenvalues and oscillator strengths on all systems. From these calculations, we report the absorbance values by using the imaginary part of the complex dielectric function.

## Supplementary information

Supplementary Information

## Data Availability

The data that support the findings of this study are available from the corresponding author upon request.
