# Supplementary material for: Controlling interlayer excitons in MoS2 layers grown by chemical vapor deposition
Source: Nat Commun. 2020 May 13;11:2391. doi: 10.1038/s41467-020-16023-z (PMC7220905; doi:10.1038/s41467-020-16023-z)
Supplement: Supplementary file 1 — Supplementary Information [file 41467_2020_16023_MOESM1_ESM.pdf]

Controlling interlayer excitons in MoS_2 layers grown by chemical vapor deposition

Supplementary Information (SI)

Ioannis Paradisanos[1*], Shivangi Shree[1*], Antony George[2], Nadine Leisgang[3], Cedric Robert[1], Kenji Watanabe[4], Takashi Taniguchi[4], Richard Warburton[3], Andrey Turchanin[2,5], Xavier Marie[1], Iann Gerber[1], and Bernhard Urbaszek[1]
[1] *Université de Toulouse, INSA-CNRS-UPS, LPCNO, 135 Avenue Rangueil, 31077 Toulouse, France*
[2] *Friedrich Schiller University Jena, Institute of Physical Chemistry, 07743 Jena, Germany*
[3] *Department of Physics, University of Basel, Basel, Switzerland*
[4] *National Institute for Materials Science, Tsukuba, Ibaraki 305-0044, Japan and*
[5] *Abbe Centre of Photonics, 07745 Jena, Germany*

Second harmonic generation (SHG) spectroscopy, which is a second-order nonlinear process, has been performed to determine the stacking order of CVD grown MoS_2 bilayers. Broken inversion symmetry and strong light matter interaction in bilayer 3R MoS_2 enables us to observe higher harmonics generation. In a SHG experiment, two photons of the same frequency are converted into a single photon with twice that frequency:

$$\mathbf{P}_i^{(2)}(2\omega) \propto \chi_{ijk}^{(2)} \mathbf{E}_i(\omega)\mathbf{E}_k(\omega) \quad (1)$$

where, $\mathbf{P}_i$ is the induced polarization and $\mathbf{E}_{i,k}$ the applied electric field vector components. TMD monolayer crystal lattices in the 2H phase belong to the trigonal prismatic (D_{3h}) point group. The second-order nonlinear susceptibility tensor $\chi_{ijk}^{(2)}$ has four non-zero elements with one free parameter $\chi_0^{(2)}$ in this symmetry [1, 2]. The TMD layers are excited with linearly polarized light under normal incidence. We detect the SHG response with a linear polarizer, oriented along the fundamental polarization angle. This results in a six-fold SHG intensity pattern $I_{\mathrm{SHG}}^{\parallel} \propto \cos^2 3(\theta - \theta_0)$ (SHG polarization parallel to the excitation polarization), where the angle θ_0 is the rotation of the armchair direction of the crystal relative to the laboratory axis. Polarization-resolved SHG experiments were performed using a home-built, confocal microscope set-up at room temperature. A Ti:Sapphire laser source with 76 MHz repetition rate at a wavelength of 804 nm was used. The laser (average power 8 mW) with a spot size ($\sim$ 1.5 μm) was focused on the sample by a microscope objective lens ($NA = 0.65$) at normal incidence and with a fixed linear polarization. The SHG signal was collected by the same objective and directed through a dichroic beamsplitter to a spectrometer with a 300 grooves/mm grating and a nitrogen cooled silicon charge-coupled device (CCD). The SHG intensity strongly depends on the polarization angle (θ - θ_0) between the laser polarization $E(\omega)$ and the armchair direction of the crystal shown in Supplementary Figure 1. To perform polarization-resolved SHG, the laser polarization was rotated using a half-wave plate, and the SHG intensity $I_{\mathrm{SHG}}^{\parallel}$ was collected. The polar plot of 3R MoS_2 bilayer in Supplementary Figure 2 directly reveals the stacking order and the crystallographic orientation of the bilayer. It is important to note that for the 2H MoS_2 bilayers no SHG signal at all could be detected as inversion symmetry is restored. SHG spectroscopy therefore allows a clear distinction between 3R and 2H stacked bilayers studied in this work.

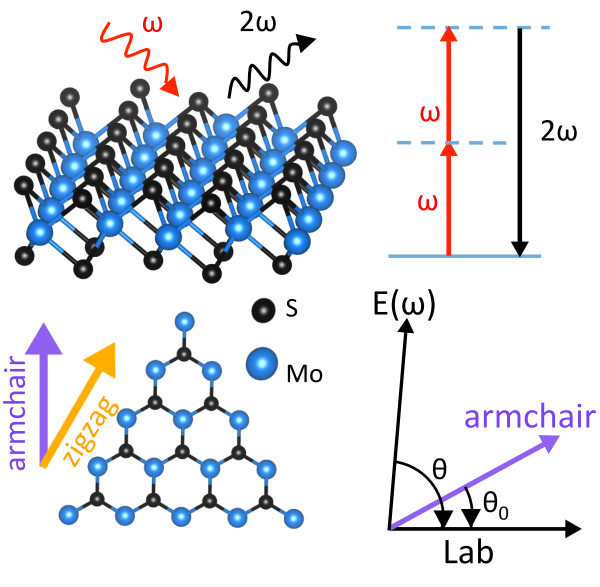

Supplementary Figure 1: Schematic representation of the crystallographic orientation of MoS_2 monolayer and the polarization of the SHG.

Photoluminescence (PL) spectroscopy has been employed to evaluate the optical quality of the individual MoS_2 MLs after the water-assisted transfer. The PL experiments were carried out in a confocal microscope built in a vibration free, closed cycle cryostat from Attocube at T = 4 K. The excitation/detection spot diameter of the 532 nm laser is below 1μm. The optical signal is dispersed in a spectrometer and detected with a Si-CCD camera. In Supplementary Figure 3 we present a typical PL spectrum collected from an hBN-encapsulated ML MoS_2 next to the manually assembled bilayers. The A-exciton emission has a linewidth of 8 meV at the full

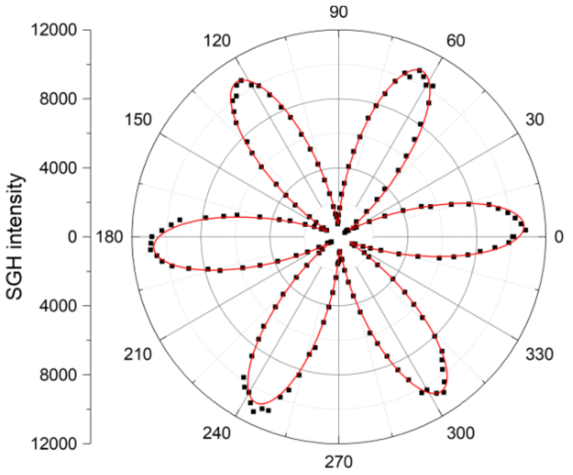

Supplementary Figure 2: Polar plot of the polarized SHG signal of CVD grown 3R MoS_2 bilayer.

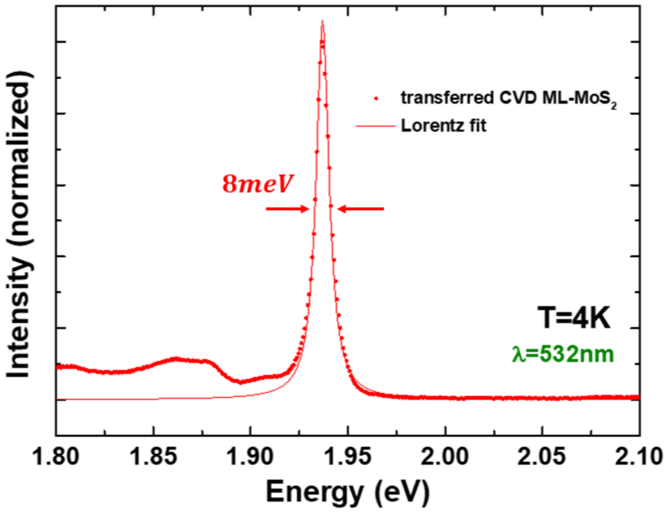

Supplementary Figure 3: PL spectrum of ML MoS_2 transferred and encapsulated in hBN following the water-assisted transfer method. A laser (532 nm) was used as an excitation source and the spectrum was collected at $T = 4$ K.

width at half maximum (FWHM), very close to the recently reported high optical quality encapsulated CVD ML MoS_2 [3].

Clear observation of interlayer excitons for CVD grown samples is due to the improved optical quality as compared to earlier studies and due to performing the measurements at cryogenic temperatures. Temperature (i.e. thermal effects), contamination of sample-substrate interface, interference effects and intrinsic sample quality are factors that may have a detrimental contribution on the detection of interlayer excitons in differential reflectivity experiments. The spectral linewidth and the exciton oscillator strength are highly sensitive to the aforementioned factors. Encapsulation of as-grown and manually-assembled CVD bilayers combined with a careful selection of the top and bottom hBN thickness significantly reduces the inhomogeneous broadening and maximizes the oscillator strength of interlayer excitons. This results in high optical quality samples for a precise determination of the interlayer interaction in different stacking orders.

In the main text we only discuss the conduction and valence states around the K-point of the Brillouin zone, here we show in Supplementary Figure 4 the band structure calculations over the full Brillouin zone using Γ-M-K-Γ path of 2H and 3R bilayers at the G_0W_0 level of theory. The degeneracy leave that appears when going from the 2H to the 3R case is present almost over the entire path.

Supplementary References

[1] Mennel, L., Paur, M. & Mueller, T. Second harmonic generation in strained transition metal dichalcogenide monolayers: Mos2, mose2, ws2, and wse2. *APL Photonics* **4**, 034404 (2019).

[2] Leisgang, N. *et al.* Optical second harmonic generation in encapsulated single-layer inse. *AIP Advances* **8**, 105120 (2018).

[3] Shree, S. *et al.* High optical quality of mos2 monolayers grown by chemical vapor deposition. *2D Materials* **7**, 015011 (2019).

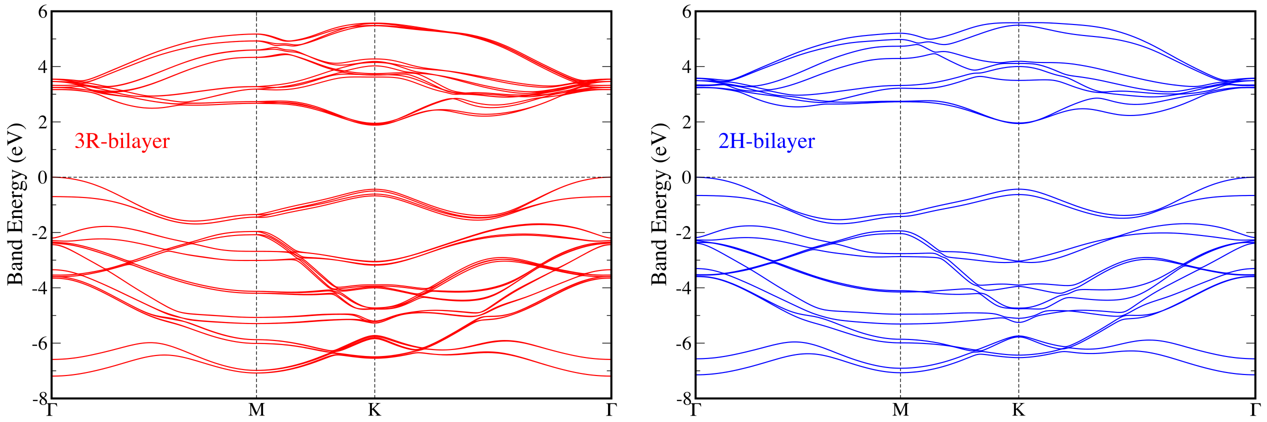

Supplementary Figure 4: G_0W_0 band structures of 2H and 3R bilayers.

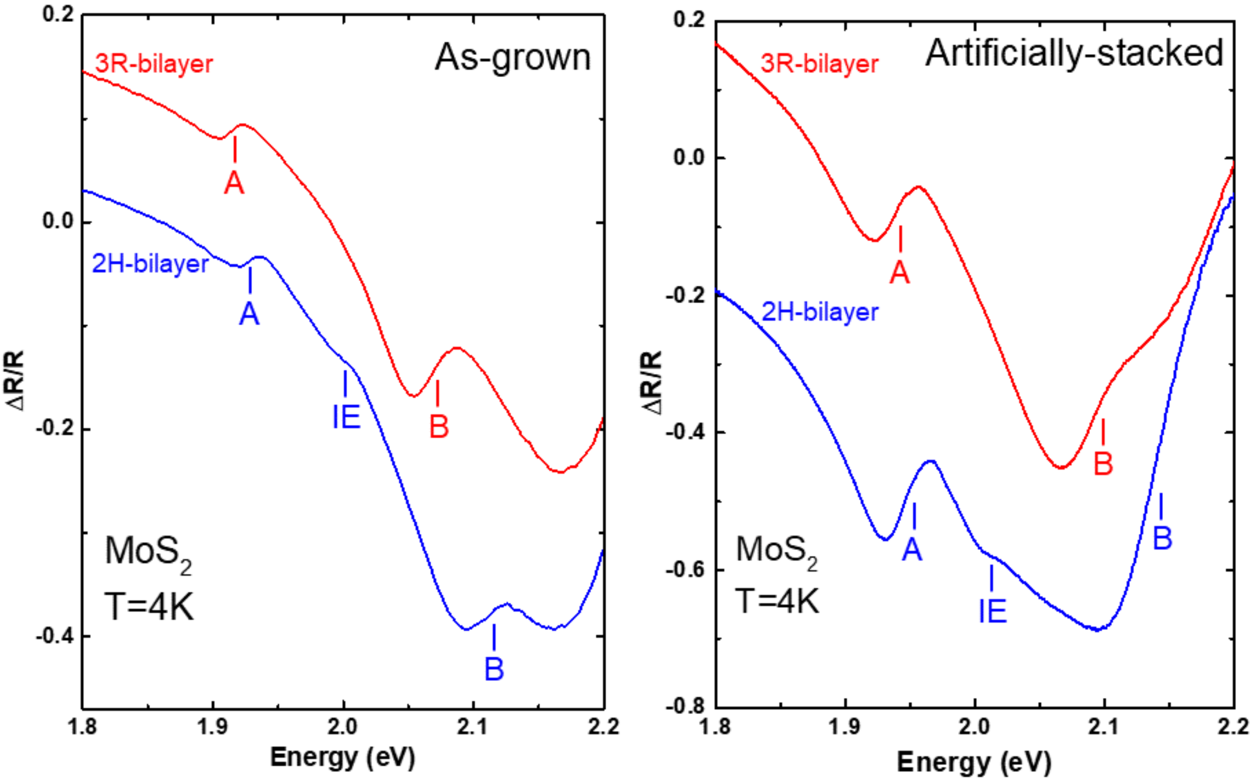

Supplementary Figure 5: In the main text we show the first derivative of the differential reflectivity. Here we show the differential reflectivity where the same main features and energy separations can be observed. This data is from the same sample and the same sample position as the in the main text.
